# Applications of artificial intelligence in ultrasound imaging for carpal-tunnel syndrome diagnosis: a scoping review

**DOI:** 10.1007/s00264-025-06497-1

**Published:** 2025-03-18

**Authors:** Yosra Magdi Mekki, Hye Chang Rhim, Daniel Daneshvar, Antonios N. Pouliopoulos, Catherine Curtin, Elisabet Hagert

**Affiliations:** 1 College of Medicine, Qatar University,, Doha, Qatar; 2 Department of Physical Medicine and Rehabilitation, Harvard Medical School, Spaulding Rehabilitation Hospital, Boston, MA USA; 3 Department of Surgical & Interventional Engineering, School of Biomedical Engineering & Imaging Sciences, King’s College London, London, UK; 4 Department of Plastic Surgery, Stanford Medicine, Stanford, CA USA; 5 Aspetar Orthopedic and Sports Medicine Hospital, Doha, Qatar; 6 Karolinska Institutet, Stockholm, Sweden

**Keywords:** Carpal tunnel syndrome (CTS), US imaging, Artificial intelligence (AI), Scoping review

## Abstract

**Purpose:**

The purpose of this scoping review is to analyze the application of artificial intelligence (AI) in ultrasound (US) imaging for diagnosing carpal tunnel syndrome (CTS), with an aim to explore the potential of AI in enhancing diagnostic accuracy, efficiency, and patient outcomes by automating tasks, providing objective measurements, and facilitating earlier detection of CTS.

**Methods:**

We systematically searched multiple electronic databases, including Embase, PubMed, IEEE Xplore, and Scopus, to identify relevant studies published up to January 1, 2025. Studies were included if they focused on the application of AI in US imaging for CTS diagnosis. Editorials, expert opinions, conference papers, dataset publications, and studies that did not have a clear clinical application of the AI algorithm were excluded.

**Results:**

345 articles were identified, following abstract and full-text review by two independent reviewers, 18 manuscripts were included. Of these, thirteen studies were experimental studies, three were comparative studies, and one was a feasibility study. All eighteen studies shared the common objective of improving CTS diagnosis and/or initial assessment using AI, with shared aims ranging from median nerve segmentation (*n* = 12) to automated diagnosis (*n* = 9) and severity classification (*n* = 2). The majority of studies utilized deep learning approaches, particularly CNNs (*n* = 15), and some focused on radiomics features (*n* = 5) and traditional machine learning techniques.

**Conclusion:**

The integration of AI in US imaging for CTS diagnosis holds significant promise for transforming clinical practice. AI has the potential to improve diagnostic accuracy, streamline the diagnostic process, reduce variability, and ultimately lead to better patient outcomes. Further research is needed to address challenges related to dataset limitations, variability in US imaging, and ethical considerations.

**Supplementary Information:**

The online version contains supplementary material available at 10.1007/s00264-025-06497-1.

## Introduction

Carpal Tunnel Syndrome (CTS) is a prevalent entrapment neuropathy affecting approximately 3–6% of the general population [1, 2]. This condition arises from the compression of the median nerve as it passes through the carpal tunnel in the wrist. Individuals engaged in repetitive wrist activities or those with certain medical conditions like diabetes and rheumatoid arthritis are at a higher risk. CTS typically manifests as pain, numbness, and tingling in the hand and fingers, particularly affecting the thumb, index, middle, and part of the ring finger [2]. Patients may also experience weakness in grip strength and difficulty with fine motor tasks [1].

While clinical examination remains the gold-standard, the use of Ultrasound (US) has become a valuable tool in diagnosing CTS, offering a non-invasive method to visualize the median nerve and assess its morphology. It allows for real-time imaging, enabling dynamic assessment of the nerve and surrounding structures during wrist movement [3]. Although magnetic resonance imaging (MRI) is considered the best imaging test for diagnosing peripheral neuropathy, it is costly and has difficult-to-meet requirements. Computed Tomography (CT) can also be used to assess carpal tunnel anatomy; however CT exposes the patient to radiation, has limited visualization of the nerve and is expensive.

US effectively measures the cross-sectional area (CSA) of the median nerve, a key indicator of CTS. CSA technique boasts high sensitivity and specificity, making it a reliable alternative or adjunct to electrodiagnostic testing [4, 5]. US can also identify other potential causes of median nerve compression, such as cysts or anatomical variations. Segmentation, a crucial step in this process, involves isolating the median nerve to ensure accurate measurement and identification of these causes [6]. Despite its advantages, US has limitations. Its accuracy is influenced by operator experience and equipment variability [7]. It does not evaluate detect proximal causes of symptoms like cervical radiculopathy [5], and can yield false negatives in early CTS stages [8, 9].

Artificial intelligence (AI) is rapidly transforming medical imaging, offering the potential to enhance the accuracy and efficacy of image analysis [10]. This technology utilizes complex computing methods to analyze medical images, extract meaningful information, and assist healthcare professionals in diagnosis and treatment planning. Deep learning, a subfield of AI, employs artificial neural networks to mimic the human brain’s learning process [11]. These algorithms, particularly Convolutional Neural Networks (CNNs), excel at recognizing complex patterns and features within images, making them particularly well-suited for medical image analysis [12]. CNNs are capable of classifying medical images and identifying pathologies, leading to advancements in various medical specialties.

In the context of CTS, AI can automate the identification and measurement of the median nerve in US images [13, 14]. This could reduce reliance on manual interpretation, which is subjective and time-consuming, thus improving diagnostic precision. AI can analyze large datasets of US images quickly facilitating earlier detection of CTS [12, 13]. By automating tedious tasks and providing objective measurements, AI can assist clinicians in making more informed and timely diagnoses, leading to improved patient care.

This scoping review, conducted in accordance with the Arksey & O’Malley framework [15], aimed to map the literature on the application of AI techniques to US image analysis for CTS diagnosis. We examine the different AI techniques employed, their reported performance, and the challenges associated with their implementation in clinical practice. Specifically, we sought to address the following key questions covered in Table 1.

**Table 1:** Key concepts and research questions

Concept	Research questions
Artificial intelligence	• Which AI architectures have been applied to US imaging for CTS diagnosis? • What metrics are used to evaluate the diagnostic performance of AI models in CTS diagnosis? • How do the AI models’ performance compare to that of human experts or other diagnostic methods?
Carpal tunnel syndrome	• What are the key challenges and limitations in developing and implementing AI algorithms for CTS diagnosis using US? • What are the potential benefits of using AI in US for CTS diagnosis? • What are the future directions for research and development in this area?

## Methods

### Search strategy

**Fig. 1:**
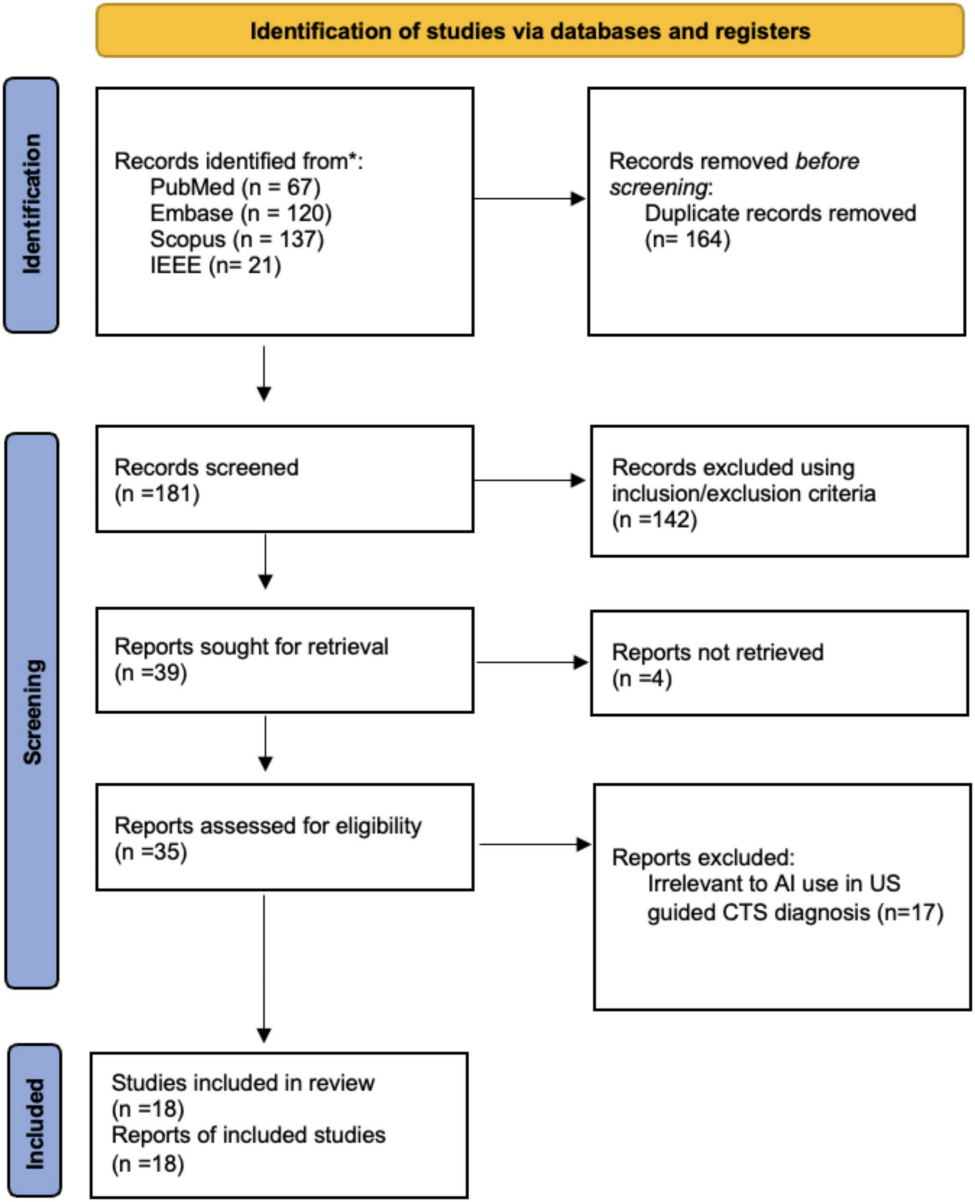
PRISMA flowchart

This scoping review was conducted following the Arksey & O’Malley framework [15] and informed by the PRISMA-ScR [16] (Preferred Reporting Items for Systematic reviews and Meta-Analyses extension for Scoping Reviews) guidelines (Fig. 1 for PRISMA Chart).

A comprehensive literature search was conducted across four electronic databases—Embase, PubMed, IEEE Xplore, and Scopus—to identify relevant studies published up to January 1, 2025. The most recent search was conducted on January 1, 2025. The following search terms were used: “carpal tunnel syndrome,” “median nerve compression,” “artificial intelligence,” “machine learning,” “deep learning,” “neural networks,” and “computer-aided diagnosis.” See Table 2 for a detailed keyword strategy for each database.

**Table 2:** Search strategy summary to maintain a focused scope on AI in US imaging for CTS diagnosis, certain publications were excluded. These include editorials, expert opinions, conference papers, and dataset publications that lack significant analysis of AI applications. Additionally, studies without a clear clinical application of the AI algorithm or not focused on AI for US-based CTS diagnosis were excluded. See Table 3 for the inclusion/exclusion criteria

Database	Search String	Returned results	Last search date
Embase	(‘carpal tunnel syndrome’/exp OR ‘carpal tunnel syndrome’:ti, ab OR ‘median nerve compression’:ti, ab) AND (‘artificial intelligence’/exp OR ‘artificial intelligence’:ti, ab OR ‘machine learning’/exp OR ‘machine learning’:ti, ab OR ‘deep learning’:ti, ab OR ‘neural networks’:ti, ab OR ‘computer aided diagnosis’:ti, ab)	**120**	**1/1/25**
Scopus	( TITLE-ABS-KEY ( “carpal tunnel syndrome” OR “median nerve compression” ) AND ( TITLE-ABS-KEY ( “artificial intelligence” OR “machine learning” OR “deep learning” OR “neural networks” OR “computer aided diagnosis” ) ) )	**137**	**1/1/25**
IEEEXplore	((“carpal tunnel syndrome”) OR (“median nerve compression”)) AND ((“artificial intelligence”) OR (“machine learning”) OR (“deep learning”) OR (“neural networks”) OR (“computer-aided diagnosis”))	**21**	**1/1/25**
Pubmed	(“carpal tunnel syndrome“[MeSH Terms] OR “carpal tunnel syndrome“[Title/Abstract] OR “median nerve compression“[Title/Abstract]) AND (“artificial intelligence“[MeSH Terms] OR “artificial intelligence“[Title/Abstract] OR “machine learning“[MeSH Terms] OR “machine learning“[Title/Abstract] OR “deep learning“[Title/Abstract] OR “neural networks“[Title/Abstract] OR “computer-aided diagnosis“[Title/Abstract])	**67**	**1/1/25**

**Table 3:** Inclusion and exclusion criteria

Criterion	Inclusion	Exclusion
Topic	Studies applying AI, ML, or DL to diagnose, predict, or manage carpal tunnel syndrome using US	Editorials, expert opinions, conference papers, dataset publications.
Methodology	Original articles with algorithms tested and trained on paediatric orthopaedic datasets. Algorithms must utilize advanced AI methods with explainable/interpretable results.	Studies with no clear clinical application of algorithm and dataset publications.
Outcomes	Clinical focus on accuracy, prediction, decision-making, or improvement	Studies not focused on AI application in US and carpal tunnel syndrome
Publication	Peer-reviewed, published in English	Outdated algorithms.

The search strategies were adapted for each database using appropriate syntax and subject headings (where applicable). After removing duplicates using Rayyan, 181 articles remained. Two independent reviewers conducted the full-text review and screening process. These articles were screened based on their titles and abstracts, resulting in 39 articles for full-text review. Of these, 17 were excluded due to their focus on other imaging modalities, or unavailability of full-text in online repositories (*n* = 4). Any disagreements between reviewers were resolved by a third reviewer. As this is a scoping review, a formal quality assessment of the included articles was not performed. The review focused solely on published studies, excluding grey literature.

### Data extraction (Data charting)

Data extraction was performed on the 18 included articles by 2 independent reviewers. Key study information was gathered, including author details, publication year, study objectives, and study design. Specific details regarding the US imaging techniques employed were also recorded, such as the US frequencies used, specific settings, and measurement techniques (e.g., CSA of the median nerve, nerve mobility). Patient characteristics where available, including age, severity of CTS, and occupation, were documented. To assess AI performance, information about comparator groups was extracted, including the expertise level of human comparators (if applicable). The data extraction process also examined the specifics of the AI algorithms, such as the architecture used, annotator expertise, workflow, training set demographics, validation techniques, and model output. Finally, the outcome parameters used to evaluate the AI models were recorded, including metrics like sensitivity, specificity, accuracy, and area under the receiver operating characteristic curve (AUC). The supplementary tables (Tables 4, 5, 6 and 7) detail the parameters of data extraction for each evidence source.

## Results

### Search results and study characteristics

In accordance with the PRISMA-ScR charting [16] requirements, Tables 4, 5, 6 and 7 present the characteristics of included studies and their extracted data. Table 4 summarizes the study characteristics included in this review. The studies included in this review were published between 2020 and 2024, with increased volume over time (two studies in 2020 to six studies in 2024). Regarding study design, thirteen studies were experimental studies, three were comparative studies, and one was a feasibility study. The studies were from many countries with the most publications from China and Japan. While the specific methodologies varied, all eighteen studies shared the common objective of improving CTS diagnosis and/or initial assessment using AI, and most shared the aims of median nerve segmentation (*n* = 12) and automated diagnosis (*n* = 9). The majority of studies utilized deep learning approaches, particularly CNNs (*n* = 15).

Seven studies focused on the median nerve at the carpal tunnel inlet or proximal inlet. Other anatomical locations also evaluated. (Table 5). All studies utilized specific settings and techniques such as measuring the CSA of the median nerve.

Ten studies used human experts, such as radiologists or sonographers, to compare against the AI’s performance (Table 6). All annotators who provided the ground truth data for training and evaluating the AI models were clinical experts in related disciplines. The workflow for most studies (*n* = 17) involved acquiring US images, manually annotating the median nerve, training the AI model, and then evaluating its performance on a separate dataset.

Seven studies included both CTS patients and healthy controls in their training sets, while six studies focused only on CTS patients (Table 7). For validation techniques, two studies used cross-validation while fourteen used separate testing sets. Regarding model outputs, five studies produced segmentation masks of the median nerve, with four of these also calculating CSA and three including perimeter measurements. For outcome parameters, ten studies reported accuracy metrics, and four studies used the Dice similarity coefficient.

### CTS diagnosis, classification and nerve tracking

Deep learning models were applied to automate CTS diagnosis, assess severity in ultrasound imaging, and automate nerve tracking.

Shinohara et al. trained an AI model on 10,000 US images from 100 patients (50 CTS wrists, 50 normal). Shinohara et al. achieved 95.9% accuracy and 99.7% specificity [17]. In another study, Lyu et al. used a random forest model with radiomic features to assess CTS severity, achieving 100% accuracy in training data but 76.39% accuracy on testing data, showing which shows that the model might need more generalizable data to improve reliability in different settings [18].

Waki et al. [19] developed an AI model that classified CTS severity with Bland’s classification for CTS severity [20] using video datasets. Their model automatically segmented the median nerve from video datasets, achieved 75% accuracy. Peng et al. [21] proposed a fully automated CTS diagnostic system, combining segmentation with nerve morphology analysis. This system achieved 93.85% accuracy, 85.00% sensitivity, and 97.78% specificity.

Tanaka et al. tested a model called YOLOv5, which is good at detecting objects, to track the median nerve’s movement during finger flexion and extension in dynamic ultrasound images [22]. Their best-performing model achieved high precision (0.953) and recall (0.956), meaning it accurately identified the nerve in most cases and didn’t miss many. Gujurati et al. used a different type of AI called transformer-based models to identify the median nerve in ultrasound videos. Their model showed nearly 94% agreement for images taken at the wrist and 84% agreement for images taken in the forearm, indicating strong performance in identifying the nerve in these areas [23].

### Median nerve segmentation and classification

AI is increasingly being used to automate the identification and outlining (segmentation) of the median nerve in US imaging.

Six studies focused on automating this segmentation process using deep learning models [6, 23–27]. In three of these studies, the AI was able to process the US images without any manual preparation, making the process faster and more efficient [13, 19, 25]. The most common AI models used were U-Net (*n* = 7), Mask R-CNN (*n* = 3), and DeepLabv3+ (*n* = 2).

Segmentation accuracy is assessed by comparing AI-generated images to expert manual segmentations using two key metrics: the Dice Similarity Coefficient (DSC), which measures how closely AI-generated and expert-segmented images overlap (where 1.0 = perfect match); and the Intersection over Union (IoU), which measures the percentage of agreement between AI and manual segmentations. For instance, Yeh et al. used a modified SOLOv2 model and reported a high DSC of 0.922 and an IoU of 0.855 [27], showing a high level of agreement between the AI and expert segmentations.

In addition to segmentation, AI can automate the measurement of median nerve parameters, such as CSA, and to analyze nerve movement. Ando et al. found that AI measurements of the median nerve were very close to manual measurements, with an error of only 0.92 mm², and a high level of agreement between the two methods (0.97 on a scale from 0 to 1) [14]. The study used 600 median nerve images, split into training (450 images), validation (50 images), and testing (100 images) sets, with the training data being augmented to 900 images. Data was collected from clinical studies where patients with CTS and healthy volunteers underwent standardized US exams, with images captured during finger movement to observe changes in the nerve.

### Radiomics in median nerve imaging

Radiomics is an advanced technique used in medical imaging to extract a wide range of quantitative features from images, like texture, shape, and intensity patterns [18]. In the case of median nerve imaging, radiomics helps to analyze the nerve’s structure by processing ultrasound (US) images.

Five studies used radiomics combined with machine learning models [18, 24, 28–30]. DeepNerve [24] was able to automatically identify the median nerve in dynamic US images, achieving 99.7% accuracy, a high recall rate of 91.19% (showing it could identify most cases), and 89.12% precision (indicating a low number of false positives). It also calculated several features of the median nerve, such as area, perimeter, shape ratio, and circularity, which help describe the nerve’s structure.

Kim et al. used AI to analyze muscle US images, focusing on the thenar and hypothenar muscles, and achieved a strong performance score of 0.89 [30]. This model extracted 176 features from the US images, providing detailed insights into muscle and nerve characteristics. Other studies also combined radiomics with machine learning. For instance, one study using Support Vector Machine classifiers reported a performance score of 0.926, while another used deep learning models and achieved scores of 0.910 and 0.908, showing strong overall performance in detecting nerve and muscle features [29].

### Comparison with human experts and clinical translation

Faeghi et al. [28] reported that a computer-aided diagnosis (CAD) system using these radiomic features achieved a performance score of 0.926 (where 1.0 is a perfect score), outperforming two musculoskeletal radiologists with 14 years of experience. The radiologists’ scores ranged from 0.658 to 0.736. The radiologists assessed the echogenicity (brightness) of median nerves using two methods: one looking for a honey-comb pattern and the other counting the number of fascicular nerve bundles.

The potential for clinical translation was a recurring theme, with four studies emphasizing AI’s capacity to reduce operator dependency and improve time efficiency [6, 13, 21, 31]. When compared to nerve conduction studies (NCS), AI models showed promise, potentially decreasing reliance on NCS [19]. However, challenges were noted, such as anatomical variations and the need for more diverse, multi-center datasets encompassing various CTS types [6, 13, 19, 25, 29].

## Discussion

This scoping review presents a map the landscape of AI applications in US diagnosis of CTS and shows both the considerable promise and the inherent challenges of this evolving field.

A primary theme emerging from the reviewed studies is the potential for AI to enhance the accuracy and efficiency of median nerve segmentation [6, 23–27], a crucial step in CSA estimation for diagnosis. We discussed studies that demonstrated their ability to accurately delineate the median nerve [14, 18, 23, 24, 28–30]. The use of these tools may lead to earlier diagnosis, allowing for timely intervention and potentially preventing further nerve damage. Precise CSA measurements and AI-driven analysis can ease barriers to patient diagnosis of this condition.

Another key finding is the ability of AI models to achieve diagnostic accuracy comparable to experienced radiologists [28]. This suggests that AI could serve as a valuable tool for augmenting clinician expertise, potentially reducing diagnostic errors and improving patient outcomes. This is particularly relevant given the inter-observer variability inherent in manual US interpretation. This could potentially improve access to this care alleviating healthcare system burdens.

Beyond segmentation and diagnosis, AI is being explored for automated measurement of median nerve parameters (CSA, movement, shape), with strong agreement between AI-driven CSA measurements and manual measurements [17]. This demonstrates the potential for objective and reliable assessment of nerve morphology. Furthermore, AI is being applied to analyze nerve movement [22], offering insights into altered nerve mechanics in CTS, potentially providing a more comprehensive understanding of the condition.

### Addressing challenges and limitations

This scoping review has inherent limitations. As a scoping review, it aimed to map the literature, not assess study quality. Despite a comprehensive search, some relevant studies, especially grey literature, may have been missed. The focus on English-language publications introduces potential language bias. Finally, the rapid evolution of AI means these findings represent a snapshot of the current landscape.

Despite promising advancements, several challenges must be addressed before AI can be fully integrated into routine CTS diagnosis and assessment. A major problem is the need for large, diverse, and well-annotated datasets [32]. As highlighted in this review, the regional concentration of research (majority Asian datasets) raises concerns about the generalizability of these AI models (for e.g. for African patients). The operator-dependent nature of US imaging introduces variability in image quality, further complicating model training [5]. Standardized imaging protocols are crucial to mitigate this variability, but implementing such protocols across different clinical settings can be challenging. The risk of bias in healthcare AI is influenced by factors such as data quality, operator variability, and demographic disparities, which can impact the accuracy and fairness of clinical decision-making [33]. In ultrasound imaging, for instance, operator-dependent variability in image quality complicates model training, making it crucial to implement standardized imaging protocols, though achieving consistency across clinical settings can be challenging [5].

A primary challenge lies in the inherent opacity of certain AI algorithms, often referred to as the “black box” problem [32]. Addressing this issue through the development and implementation of explainable AI (XAI) is critical for creating trust and ensuring clinical acceptance [34]. Practitioners require insight into the decision-making processes of these systems before they can confidently incorporate AI-driven insights into patient care. Intuitive user interface design and robust training programs for clinicians are essential for the effective utilization of AI tools [35]. Beyond usability, ethical considerations, including the protection of patient privacy, the mitigation of algorithmic bias, and the establishment of clear lines of responsibility and accountability, must be rigorously addressed [36]. The appropriate calibration between AI assistance and human oversight is vital to prevent over-reliance and preserve the essential role of clinical judgment.

## Conclusion and future directions

AI, particularly deep learning models like CNNs and transformers, offers promising avenues for enhancing various facets of CTS diagnosis, from automated nerve segmentation and objective parameter measurement to improved diagnostic accuracy and severity classification. The development of integrated diagnostic systems, combining AI with complementary imaging modalities, represents a particularly exciting direction for future exploration.

We observed a diverse range of AI architectures being employed, with CNNs and their variants currently dominant, and growing interest in transformer-based models. Performance evaluation relies on a spectrum of metrics, including accuracy, sensitivity, specificity, AUC, DSC, and IoU, reflecting the multifaceted nature of diagnostic assessment. Persistent challenges related to dataset limitations, generalizability, and explainability must be addressed.

The concentration of research within specific populations may increase the risk of bias and limits the generalizability of existing models. Standardized imaging protocols are crucial for minimizing variability and ensuring consistency across clinical settings. Furthermore, the “black box” nature of many AI algorithms necessitates the development of methods that enhance transparency and explainability, increasing trust among clinicians and facilitating informed decision-making. Ethical considerations, including data privacy and security, must also be prioritized.

Future research should prioritize the development of robust, generalizable, and ethically sound AI models, focusing on data diversity, algorithm transparency, and seamless integration into clinical practice. This includes exploring strategies for data augmentation and synthetic data generation, developing explainable AI (XAI) techniques, and designing user-friendly interfaces that facilitate clinician adoption. Multi-center studies involving diverse patient populations are crucial for validating model performance and ensuring generalizability. Ultimately, the successful translation of AI into the clinic will depend on collaborative efforts between researchers, clinicians, and regulatory bodies to ensure that these powerful tools are used responsibly and effectively to improve the diagnosis and management of CTS.

## Electronic supplementary material

Below is the link to the electronic supplementary material.

Supplementary Material 1

## Data Availability

No datasets were generated or analysed during the current study.
