# Supplementary material for: Applications of artificial intelligence in ultrasound imaging for carpal-tunnel syndrome diagnosis: a scoping review
Source: Int Orthop. 2025 Mar 18;49(4):965–73. doi: 10.1007/s00264-025-06497-1 (PMC11971218; doi:10.1007/s00264-025-06497-1)
Supplement: Supplementary file 1 — Supplementary Material 1 [file 264_2025_6497_MOESM1_ESM.docx]

**Table 4- Research Study Characteristics**

| **Author(s)** | **Year/Country** | **Study Objective** | **Study Design** |
| --- | --- | --- | --- |
| Smerilli et al.[13] | 2022, Italy | Develop CNN for median nerve identification/measurement in US for CTS diagnosis | Experimental: CNN for median nerve identification/measurement |
| Horng et al.[24] | 2020, Taiwan | Propose CNN (DeepNerve) for median nerve localization/segmentation for CTS accuracy | Experimental: CNN (DeepNerve) development for localization/segmentation in US sequences |
| Shinohara et al.[17] | 2022, Japan | Utilize deep learning for accurate CTS diagnosis from US images | Experimental: Deep learning for CTS diagnosis from US images |
| Tanaka et al.[22] | 2023, Japan | Analyze median nerve dynamics using YOLOv5 for improved CTS diagnosis | Experimental: YOLOv5 model for dynamic median nerve analysis |
| Kim et al.[30] | 2023, S. Korea | Assess feasibility of AI-assisted quantitative muscle US for CTS | Feasibility: AI-assisted quantitative muscle US for CTS |
| Wu et al.[25] | 2021, China | Evaluate deep learning model for automated median nerve segmentation in dynamic US | Experimental: Deep learning model evaluation for automated segmentation in dynamic US |
| Ando et al.[14] | 2024, Japan | Investigate CNNs for median nerve morphological assessment from US | Experimental: CNNs for morphological assessment from US images |
| Mohammadi et al.[29] | 2023, Iran | Explore deep radiomics features for automated CTS diagnosis using US | Multi-center: Deep radiomics features for automated CTS diagnosis |
| Moser et al.[6] | 2024, Germany | Train U-Net for median nerve recognition/measurement in US for CTS diagnosis | Experimental: U-Net training for median nerve segmentation in US images |
| Cosmo et al.[31] | 2022, Italy | Propose automatic deep learning for median nerve segmentation in US | Experimental: Automatic deep learning for median nerve evaluation in US images |
| Waki et al.[19] | 2024, Japan | Evaluate video datasets for CTS severity classification using machine learning | Comparative: Video datasets for machine learning classification of CTS severity |
| Ardakani et al.[36] | 2020, Iran | Compare AI and other techniques (SWE, morphometry) for CTS diagnosis | Comparative: AI, shear wave elastography, morphometry for CTS diagnosis |
| Yeh et al.[27] | 2023, Taiwan | Develop real-time automated median nerve segmentation in dynamic US DL | Experimental: Real-time automated deep learning segmentation in dynamic US |
| Faeghi et al.[28] | 2021, Iran | Compare automated radiomics diagnosis with radiologists for CTS | Comparative: Automated radiomics vs. radiologists' assessment for CTS |
| Peng et al.[21] | 2024, China | Create automated diagnostic system for CTS using deep learning in US | Experimental: Automated deep learning diagnostic system for CTS |
| Lyu et al.[18] | 2024, China | Apply radiomics model to predict CTS severity based on US features | Experimental: Radiomics model for CTS severity prediction |
| Shao et al.[26] | 2022, China | Apply improved U2-Net for US median nerve image segmentation | Experimental: Improved U2-Net for median nerve image segmentation |
| Gujarati et al.[23] | 2024, USA | Develop transformer-based model for automated median nerve segmentation in video | Experimental: Transformer-based model for automated segmentation in US videos |

**Table 5- Data Charting: Study and US Details**

| **Study** | **Anatomical Location** | **US Technique** | **Comparator Group/Models** |
| --- | --- | --- | --- |
| Smerilli et al., 2022[13] | Proximal inlet of the carpal tunnel | Transverse US images, 6–18-MHz linear probe, 2017 EULAR procedures, CSA measurement | Manual measurements by expert sonographers (ICC) |
| Horng et al., 2020[24] | Wrist, carpal tunnel | ACUSON S2000, 18 L6 HD transducer, 18-MHz, right hand flat and upward | U-Net, U-Net +MaskTrack, ConvLSTM+U-Net +MaskTrack, active contour model |
| Shinohara et al., 2022[17] | Short-axis image of the median nerve at the carpal tunnel inlet | 18MHz linear probe, constant gain, dynamic range, frame rate, probe within 20mm of distal wrist crease | SqueezeNet, MobileNet_v2, EfficientNet, manual CSA measurements |
| Tanaka et al. 2023[22] | Proximal to the carpal tunnel at the wrist crease | 18 MHz linear probe, short-axis images, finger flexion and extension | 20 control hands (no CTS, normal NCS) vs. 20 CTS hands (post-surgery) |
| Kim et al., 2023[30] | Thenar and hypothenar muscles, midpoint of first/fifth metatarsal bone | HD15 system, linear array transducer, constant gain, dynamic range, depth, frequency | Hands with CTS (confirmed by EMG) vs. control hands (unaffected hands of CTS patients) |
| Wu et al., 2021[25] | Median nerve at the wrist, proximal inlet | 13–18 MHz linear transducer, dynamic images, finger flexion and extension, 38 fps video clips | DeepLabv3+, U-Net, Feature Pyramid Network, Mask R-CNN, manual annotations |
| Ando, 2024[14] | Proximal carpal tunnel | LOGIQ e US system, 12 L-RS transducer, 12 MHz, aligned with distal wrist crease, proximal edge of pisiform bone reference | Manual annotation, U-Net, SegNet |
| Mohammadi et al., 2023[29] | Carpal tunnel inlet | Institutional guidelines, perpendicular probe, no pressure, blinded sonographers, two US machines | Two radiologists (echogenicity, honeycomb pattern), nine machine learning classifiers |
| Moser et al. 2024[6] | Median nerve, distal half of forearm, carpal tunnel inlet | GE Logiq 10, 6–15 MHz probe, MSK mode, 3.5–4.0 cm depth, three video loops | 25 CTS hands vs. 26 healthy controls, manual CSA by two operators |
| Cosmo et al., 2022[31] | Proximal carpal tunnel inlet | MyLab Class C (Esaote SpA), 6–18 MHz linear probe, 2017 EULAR procedures | U-Net based models, manual measurements by sonographers |
| Waki et al. 2024[19] | Inlet of the carpal tunnel | LOGIQ e10, 24 MHz linear probe, 90° tilt, 30 fps videos (reduced to 10 fps) | Control group, mild CTS group (grades 1-3), severe CTS group (grades 4-6), models trained with different datasets |
| Ardakani et al., 2020[36] | Carpal tunnel | AixPlorer, 5-18 MHz linear array transducer, wrist extended 30-45°, forearm supinated, shear wave elastography (SWE) | 100 CTS wrists vs. 100 control wrists, SVM, CNN |
| Yeh et al., 2023[27] | Median nerve in carpal tunnel, fingers moving | Aplio 500, 13- to 18-MHz linear transducer, 38 FPS | SOLOv2, Mask-R-CNN, YOLACT, BlendMask, ensemble models |
| Faeghi et al., 2021[28] | Median nerve within the wrist | B-mode US | Wrists with CTS vs. control wrists, assessments by two expert radiologists |
| Peng et al., 2024[21] | Median nerve within the carpal tunnel | 5-12 MHz non-linear transducer | CTS patients vs. healthy volunteers, U-Net, DeepLabV3+, Mask R-CNN, Solov2 |
| Lyu et al., 2024[18] | Median nerve at the scaphoid-pisiform level | 7-15MHz US | Mild, moderate, severe CTS groups (based on CSA) vs. EMG results |
| Shao et al. 2022[26] | Median nerve at the level of the peas bone | Transverse 2D US, linear array probe | U-Net, Res-U-Net |
| Gujarati et al., 2024[23] | Median nerve from wrist to elbow | 7-15 MHz linear transducer, US video | U-Net, U-Net++, Siam U-Net, Attention U-Net, LSTM U-Net, Trans U-Net |

**Table 6- Data Charting: AI Model and Training**

| **Study** | **AI Architecture** | **Annotator** | **Workflow** | **Training Set Demographics** |
| --- | --- | --- | --- | --- |
| Smerilli et al., 2022[13] | Mask R-CNN (ResNet101, FPN) | G.Sa. (reviewed by expert sonographer) | Region proposals, object class prediction, bounding box refinement, mask generation | 157 images, 64 patients (rheumatic/musculoskeletal disorders), patients under 18 excluded |
| Horng et al., 2020[24] | DeepNerve (U-Net, MaskTrack, ConvLSTM) | Clinical expert | Image sequence processing, concatenation of current image with previous prediction | 24 image sequences, 6 male participants (4 normal, 2 CTS), 18-28 years old, ~420 frames/sequence (ROI cropped) |
| Shinohara et al., 2022[17] | SqueezeNet, MobileNet_v2, EfficientNet | Certified hand surgeon | Image capture, cropping (15x15mm), data augmentation, feature detection, heatmap visualization | 40 CTS hands, 40 control hands, total 100 participants (control: 22M/28W, 45.0±7.3 yrs; CTS: 19M/31W, 69.5±13.2 yrs) |
| Tanaka et al. 2023[22] | YOLOv5 | Experienced hand surgeon | Bounding box detection, center coordinate tracking | 5000 images, 50 hands (no CTS), 50 hands (CTS) |
| Kim et al., 2023[30] | Random forest, AdaBoost, SVC, XGB | Experienced rehabilitation physician | Conventional grayscale analysis, radiomic feature extraction (176), RFE | 47 CTS hands, 27 control hands |
| Wu et al., 2021[25] | DeepLabv3+, U-Net, FPN, Mask R-CNN | Physiatrist (musculoskeletal US expertise) | Dynamic image segmentation | 52 CTS subjects (36 training, 16 testing), 15215 training frames, 3410 testing frames |
| Ando, 2024[14] | U-Net, SegNet | ImageJ | Image acquisition, manual annotation, model training, output comparison | 600 images (left/right wrists, healthy), 450 training, 50 validation, 100 testing, augmented to 900 |
| Mohammadi et al., 2023[29] | SqueezeNet + SVM, SGD, KNN, Gradient Boosting, Random Forest, Naive Bayes, Logistic Regression, Adaptive Boosting, Decision Tree | Radiologists | Image preprocessing, feature extraction (SqueezeNet), feature reduction (ReliefF), classification | 416 nerves (Iran: 112 entrapped/112 normal development, 26/26 internal validation; Colombia: 70/70 external validation) |
| Moser et al. 2024[6] | U-Net | Experienced rheumatologist | Manual segmentation, model training, automated segmentation, measurement comparison | 25 CTS patients, 26 healthy controls, ~40 images/participant |
| Cosmo et al., 2022[31] | Mask R-CNN (ResNet-101, FPN, 2 transposed layers) | Sonographer (supervised by 2 sonographers) | Image acquisition, manual annotation, model training, evaluation | 246 images, 103 rheumatic patients (bifid nerves, closed vessels) |
| Waki et al. 2024[19] | Mask R-CNN, KNN | VGG Image Annotator | Video acquisition, median nerve segmentation, feature extraction, dataset creation, KNN model training | 75 individuals (52 CTS, 23 healthy), 132 videos |
| Ardakani et al., 2020[36] | SVM, CNN | Hybrid multilayer filtering, Chan-Vese method | US/SWE, median nerve segmentation, feature extraction, statistical analysis, classification | 200 wrists (100 CTS, 100 control) |
| Yeh et al., 2023[27] | SOLOv2-MN, Ensemble models | Expert | Dynamic image sequence acquisition, manual annotation, model training, output comparison | 9 normal, 59 CTS patients, 20294 annotated frames |
| Faeghi et al., 2021[28] | SVM | MSK radiologists | Median nerve contouring, radiomic feature extraction, classification | 228 wrists (65 CTS patients, 57 control) |
| Peng et al., 2024[21] | SegFormer | Experienced radiologists | US imaging, median nerve segmentation, parameter measurement, diagnosis | 40 CTS wrists, 90 healthy wrists |
| Lyu et al., 2024[18] | Random Forest (RFE) | MSK US expert | Image acquisition, median nerve outlining, radiomic feature extraction, classification | 237 CTS hands (106 mild, 68 moderate, 63 severe) |
| Shao et al. 2022[26] | Improved U2-Net | Clinicians (2 labeling, 1 auditing) | Image acquisition, manual delineation, model training, evaluation | 402 nerve images (249 CTS, 153 healthy), 320 training, 82 testing |
| Gujarati et al., 2024[23] | Modified Visual Transformer (VisTR) | Expert sonographers | US video acquisition, annotation, model training, segmentation, CSA calculation | 100 subjects (80 training, 10 validation, 10 testing) |

**Table 7- Data Charting: Validation, Output, and Outcomes**

| **Study** | **Validation/Evaluation Technique** | **Model Output** | **Outcome Parameter** |
| --- | --- | --- | --- |
| Smerilli et al., 2022[13] | Precision, recall, mAP, DSC | Location, pixel-level segmentation mask, automated CSA measurement | Segmentation accuracy, agreement between automated and manual CSA (ICC) |
| Horng et al., 2020[24] | Fourfold cross-validation | Segmentation mask, area, perimeter, aspect ratio, circularity | Localization and segmentation accuracy (accuracy, recall, precision, F-score, DSC, Hausdorff metric), deformation measurements |
| Shinohara et al., 2022[17] | Test data, confusion matrix, ROC curves | CTS presence/absence prediction, heatmap visualization | Accuracy, precision, recall, specificity, F-measure, AUC |
| Tanaka et al. 2023[22] | Test set, mAP, precision, recall | Bounding box coordinates, nerve displacement and velocity | Radial-ulnar and dorsal-palmar displacement and velocity |
| Kim et al., 2023[30] | AUC, precision, recall, F1 score | Binary CTS classification, important radiomic features | Diagnostic accuracy (AUC, precision, recall, F1) |
| Wu et al., 2021[25] | IoU | Binary mask, centroid position, circularity, perimeter, CSA | Segmentation accuracy (IoU), spatiotemporal profile, circularity and CSA changes |
| Ando, 2024[14] | Spearman’s correlation, Bland-Altman analysis | Segmented image, CSA, circumference, diameters | Segmentation accuracy (IoU), correlation and agreement between manual and CNN measurements |
| Mohammadi et al., 2023[29] | Internal/external validation (Iranian/Colombian datasets), sensitivity, specificity, accuracy, AUC | CTS/normal classification, entrapment prediction score | Diagnostic accuracy |
| Moser et al. 2024[6] | Dice score, paired t-test, Wilcoxon test, logistic regression | Segmented nerve, automated CSA measurement | Segmentation accuracy (Dice), difference between manual and automated CSA, diagnostic power |
| Cosmo et al., 2022[31] | Precision, recall, mAP, DSC, MAE | Segmentation mask, CSA | Segmentation performance (DSC), CSA accuracy (MAE) |
| Waki et al. 2024[19] | 63-fold cross-validation, sensitivity, specificity, accuracy, precision, recall, F1 score | CTS severity classification, segmentation mask, CSA, convex area, perimeter, axis lengths, aspect ratio, eccentricity, centroid coordinates | Severity classification accuracy, segmentation performance (IoU) |
| Ardakani et al., 2020[36] | AUC, sensitivity, specificity | CTS diagnosis, morphological parameters, elasticity parameters | Diagnostic accuracy, differentiation between severity grades |
| Yeh et al., 2023[27] | Average IoU, average precision, average recall, Dice coefficient, inference speed | Segmented image | Segmentation accuracy and speed |
| Faeghi et al., 2021[28] | ROC analysis, AUC, sensitivity, specificity | CTS diagnosis | Diagnostic accuracy (AUC) |
| Peng et al., 2024[21] | Dice, IoU, HD95, ASSD, chi-square test | Segmented nerve, diagnostic parameters, CTS diagnosis | Segmentation performance, diagnostic results |
| Lyu et al., 2024[18] | ROC curves, F1 values, confusion matrix | CTS severity classification | Predictive performance for severity classification |
| Shao et al. 2022[26] | Dice coefficient, pixel accuracy (PA), mean intersection over union (MIoU), average Hausdorff distance (AVD) | Segmented median nerve | Segmentation accuracy (Dice, MIoU, PA, AVD) |
| Gujarati et al., 2024[23] | Dice Similarity Coefficient (DSC), recall, precision, Hausdorff distance | Segmented median nerve, cross-sectional area (CSA) | Segmentation accuracy (DSC) |

**S 1 Table: PRISMA-ScR Checklist**

| SECTION | ITEM | PRISMA-ScR CHECKLIST ITEM | Reported in Section |
| --- | --- | --- | --- |
| TITLE | | | |
| Title | 1 | Identify the report as a scoping review. | Title page |
| ABSTRACT | | | |
| Structured summary | 2 | Provide a structured summary that includes (as applicable): background, objectives, eligibility criteria, sources of evidence, charting methods, results, and conclusions that relate to the review questions and objectives. | Abstract |
| INTRODUCTION | | | |
| Rationale | 3 | Describe the rationale for the review in the context of what is already known. Explain why the review questions/objectives lend themselves to a scoping review approach. | Introduction |
| Objectives | 4 | Provide an explicit statement of the questions and objectives being addressed with reference to their key elements (e.g., population or participants, concepts, and context) or other relevant key elements used to conceptualize the review questions and/or objectives. | Introduction |
| METHODS | | | |
| Protocol and registration | 5 | Indicate whether a review protocol exists; state if and where it can be accessed (e.g., a Web address); and if available, provide registration information, including the registration number. | N/A |
| Eligibility criteria | 6 | Specify characteristics of the sources of evidence used as eligibility criteria (e.g., years considered, language, and publication status), and provide a rationale. | Methods |
| Information sources | 7 | Describe all information sources in the search (e.g., databases with dates of coverage and contact with authors to identify additional sources), as well as the date the most recent search was executed. | Methods |
| Search | 8 | Present the full electronic search strategy for at least 1 database, including any limits used, such that it could be repeated. | Methods, Table 2 |
| Selection of sources of evidence | 9 | State the process for selecting sources of evidence (i.e., screening and eligibility) included in the scoping review. | Methods |
| Data charting process | 10 | Describe the methods of charting data from the included sources of evidence (e.g., calibrated forms or forms that have been tested by the team before their use, and whether data charting was done independently or in duplicate) and any processes for obtaining and confirming data from investigators. | Methods |
| Data items | 11 | List and define all variables for which data were sought and any assumptions and simplifications made. | Methods |
| Critical appraisal of individual sources of evidence | 12 | If done, provide a rationale for conducting a critical appraisal of included sources of evidence; describe the methods used and how this information was used in any data synthesis (if appropriate). | N/A |
| Synthesis of results | 13 | Describe the methods of handling and summarizing the data that were charted. | Methods |
| RESULTS | | | |
| Selection of sources of evidence | 14 | Give numbers of sources of evidence screened, assessed for eligibility, and included in the review, with reasons for exclusions at each stage, ideally using a flow diagram. | Methods |
| Characteristics of sources of evidence | 15 | For each source of evidence, present characteristics for which data were charted and provide the citations. | Results |
| Critical appraisal within sources of evidence | 16 | If done, present data on critical appraisal of included sources of evidence (see item 12). | N/A |
| Results of individual sources of evidence | 17 | For each included source of evidence, present the relevant data that were charted that relate to the review questions and objectives. | Results |
| Synthesis of results | 18 | Summarize and/or present the charting results as they relate to the review questions and objectives. | Results |
| DISCUSSION | | | |
| Summary of evidence | 19 | Summarize the main results (including an overview of concepts, themes, and types of evidence available), link to the review questions and objectives, and consider the relevance to key groups. | Discussion |
| Limitations | 20 | Discuss the limitations of the scoping review process. | Discussion |
| Conclusions | 21 | Provide a general interpretation of the results with respect to the review questions and objectives, as well as potential implications and/or next steps. | Conclusion |
| FUNDING | | | |
| Funding | 22 | Describe sources of funding for the included sources of evidence, as well as sources of funding for the scoping review. Describe the role of the funders of the scoping review. | N/A |
